# Undirected singing rate as a non-invasive tool for welfare monitoring in isolated male zebra finches

**DOI:** 10.1371/journal.pone.0236333

**Published:** 2020-08-10

**Authors:** Homare Yamahachi, Anja T. Zai, Ryosuke O. Tachibana, Anna E. Stepien, Diana I. Rodrigues, Sophie Cavé-Lopez, Corinna Lorenz, Ezequiel M. Arneodo, Nicolas Giret, Richard H. R. Hahnloser

**Affiliations:** 1 Institute of Neuroinformatics and Neuroscience Center Zurich, University of Zurich and ETH Zurich, Zurich, Switzerland; 2 Institut des Neurosciences Paris Saclay, UMR 9197 CNRS, Université Paris Saclay, Orsay, France; McGill University, CANADA

## Abstract

Research on the songbird zebra finch (*Taeniopygia guttata)* has advanced our behavioral, hormonal, neuronal, and genetic understanding of vocal learning. However, little is known about the impact of typical experimental manipulations on the welfare of these birds. Here we explore whether the undirected singing rate can be used as an indicator of welfare. We tested this idea by performing a post hoc analysis of singing behavior in isolated male zebra finches subjected to interactive white noise, to surgery, or to tethering. We find that the latter two experimental manipulations transiently but reliably decreased singing rates. By contraposition, we infer that a high-sustained singing rate is suggestive of successful coping or improved welfare in these experiments. Our analysis across more than 300 days of song data suggests that a singing rate above a threshold of several hundred song motifs per day implies an absence of an acute stressor or a successful coping with stress. Because singing rate can be measured in a completely automatic fashion, its observation can help to reduce experimenter bias in welfare monitoring. Because singing rate measurements are non-invasive, we expect this study to contribute to the refinement of the current welfare monitoring tools in zebra finches.

## Introduction

Songbirds, such as the zebra finch (*Taeniopygia guttata*), serve as the main animal model to study vocal learning—the ability to learn vocalization by imitation [1]. Despite a wide range of research [2–15], little is known about the impact of typical experimental manipulations on the songbird’s welfare [16–18].

When an animal is subjected to a stressful situation that challenges its homeostasis, its biological response is to cope with the threat by returning to a homeostatic state [19]. Failure to promptly cope with stress is indicative of poor welfare [20]. Thus, while performing scientific experiments, researchers should ensure animal welfare by avoiding unnecessary stress.

Ideally, welfare should be monitored using stress-free procedures and the results should be available quickly for a prompt welfare assessment. However, most welfare assessment methods in birds tend to be stressful and relatively slow. For example, the quantification of plasma corticosterone, a hormonal indicator of stress, requires animal handling and extraction of a blood sample. Although less invasive methods of stress assessment have been proposed such as to measure corticosterone in droppings, track body weight, and identify fault bars in feathers [21–25], these alternatives average out moments of acute stress and therefore provide, at best, a measure of chronic stress.

In search of a reliable, rapid, and non-invasive readout of acute stress, we investigated the relationship between putative stressors and singing rate. Numerous studies suggest that singing is an expression of positive welfare. In seasonal breeders, the act of singing outside of the breeding season is associated with a positive affective state, as indicated by the induction of the endogenous opioid-mediated reward pathways [26]. Song practice, when neither directed to a female nor used in territory defense (i.e. undirected singing), has recently been suggested to be a form of play [4]. In territorial birds, singing rate reflects body condition, motivation, parasite load, reproductive success, and territory quality [27–31]. Similarly, in social songbirds, a high undirected singing rate has been proposed to have a mate-attracting role [32], whereas stressors such as food and water deprivation [33] and wearing a heavy backpack [34] suppress singing.

We evaluated the effects of a variety of experimental manipulations on the undirected singing rate in male zebra finches. We performed a post hoc analysis of more than three million song motifs (stereotyped sequences of 3–7 syllables) from our ongoing and published work [35, 36]. Because isolation is usually a required condition to carry out well-controlled vocal learning experiments, all our analyses were carried out in isolated birds. Therefore, our work is not intended to be informative about the effects of social isolation, on which there is a growing literature [16, 37, 38]. Our work highlights a method for rapid detection of welfare-impacting stressors in individually housed birds.

## Materials and methods

### Animals and housing

All animal procedures were approved by the Veterinary Office of the Canton of Zurich, Switzerland (Project license 207/2013) in accordance with the Swiss Animal Welfare Ordinance (TSchV) and by the French Ministry of Research and the Ethical Committee Paris-Sud and Centre (CEEA N°59, project 2017–25).

We used data from 104 healthy male zebra finches (*Taeniopygia guttata*), age range 85–864 days post-hatch (dph). Birds were obtained from our breeding colonies in Zurich (Switzerland), Orsay (France), and a local supplier (Oisellerie du Temple, L'Isle-d'Abeau, France). In the colony, birds were in constant social contact with other males and had visual and auditory contact with females. On each leg, a loosely attached ring (of 2.5 mm internal diameter and 3.2 mm length) was used as an identifier. All birds were maintained on a 14/10 h day/night schedule with *ad libitum* access to water and food (seed mix, 4030 for exotics and estrildid finches, BiOMill AG, Herzogenbuchsee, Switzerland; or Premium Prestige for exotics birds, Versele-Laga, Deinze, Belgium). Data from one bird was discarded from the study because of a partial lack of accessible food in the cage (no millet was present and fresh seeds were covered by husks on 3 days). Food restriction is a putative stressor that causes a reduction in the singing rate [33]. Indeed, in this bird, we observed a low singing rate on the three days with partial food availability (377, 32, and 403 song motifs per day).

During the experiment, all birds were housed alone in a cage of dimensions 39 x 23 x 39 cm^3^ (cage type 1), except for the tethered birds that were housed in a cage of dimensions 24 x 24 x 20 cm^3^ (cage type 2), or 24 x 24 x 28 cm^3^ (cage type 3), as indicated in the Results section. All cages had wood shavings as bedding, perches of diverse sizes, a rock, and a spray millet, and were located inside a soundproof box situated in a room with controlled temperature (24.9–27°C) and humidity conditions (45.3–47.3%). All experiments were carried out during the light phase.

### Habituation and monitoring

**Fig 1:**
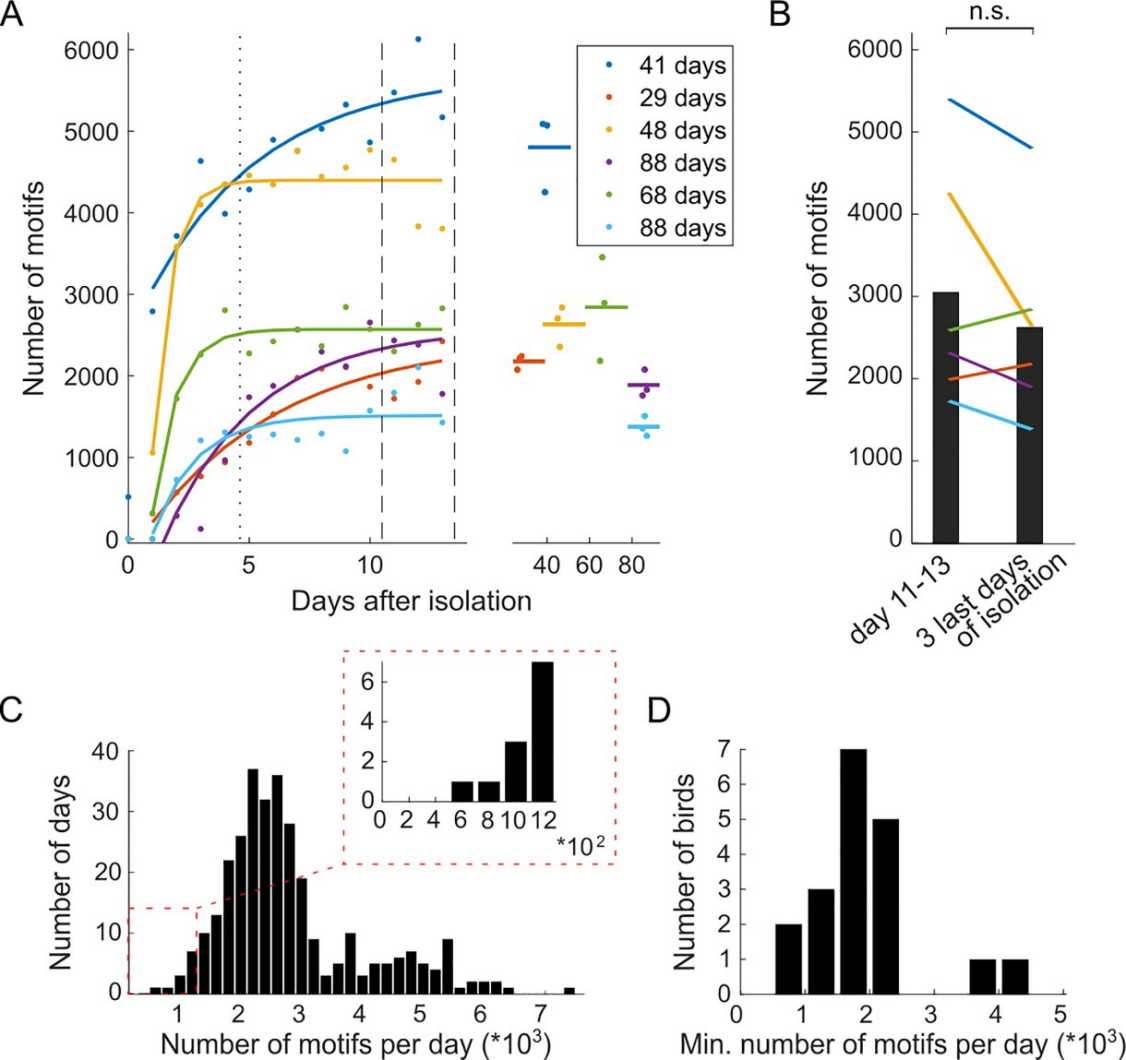
Upon isolation, the singing rate gradually increases and remains high thereafter. (A) The number of daily song motifs produced (colored dots) since the onset of social isolation on day 0. The colored dots show data from individual birds (N = 6) and the lines indicate the trend (see Methods). The dotted vertical line marks the end of the habituation period (see Results) and the dashed vertical lines delimit days 11–13 from which data was taken for panel B. The daily number of motifs produced after long-term isolation (≥ 29 days) remained high (right panel, the duration of the isolation is indicated in the legend). The horizontal lines indicate the number of motifs averaged across three consecutive days. On day zero, only recordings from after the isolation were analyzed (left panel). (B) Bar plot showing the mean motif rates across birds on days 11–13 (left) and at the end of the isolation (right). Individual lines correspond to individual birds (color matching A). (C) Histogram of the daily number of song motifs produced across 19 birds held in isolation for at least 14 days including habituation (N = 315 days after habituation). The minimum motif rate was 629 (see the zoom-in inset, note different x-axis). (D) Histogram of the minimum daily number of song motifs in those 19 birds. The minimum in each animal was computed across the 9+ days it spent in isolation after the habituation period (17±10 days, range 9–46 days).

**Fig 2:**
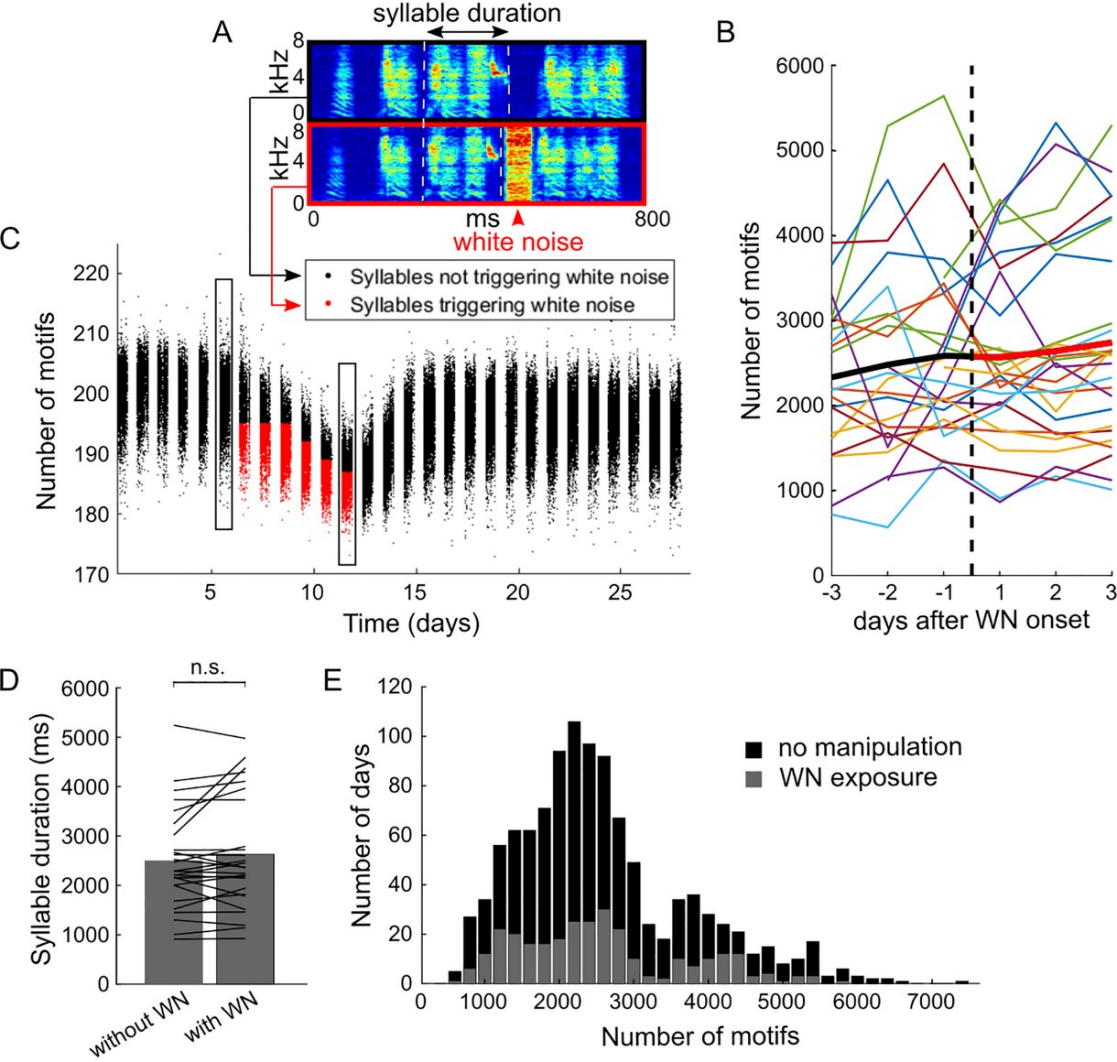
The singing rate is not affected by interactive WN. (A) Spectrograms of a song motif that did not trigger (black box) and that triggered a short burst of WN broadcasted through a loudspeaker (WN, red box). The vertical white dashed lines mark the onsets and offsets of the targeted syllable. (B) The number of motifs in each bird (colored lines) as a function of time since the onset of the interactive WN. The bold black line shows the average across birds before WN onset and the bold red line the average after WN onset (N = 26 birds, where for four birds there is partial data before WN onset). WN was triggered whenever the pitch of the targeted syllable was below (or above) a manually set threshold. (C) Example of a bird that compressed the syllable duration to trigger more noise. Syllable renditions triggering WN are marked in red, all others in black. The vertical rectangles mark the days from which the data in A were taken from. (D) Bar plot of the average daily song motif counts during days without WN (left bar) and with WN (right bar) for N = 26 birds. Lines indicate average daily song motifs in individual birds. (E) Histogram of the number of song motifs produced across all days from all N = 43 birds (including birds without manipulation, Fig 1C), where days without any manipulation are indicated in black (793 days from N = 43 birds) and days with WN are shown in dark gray (294 days from N = 26 birds).

**Fig 3:**
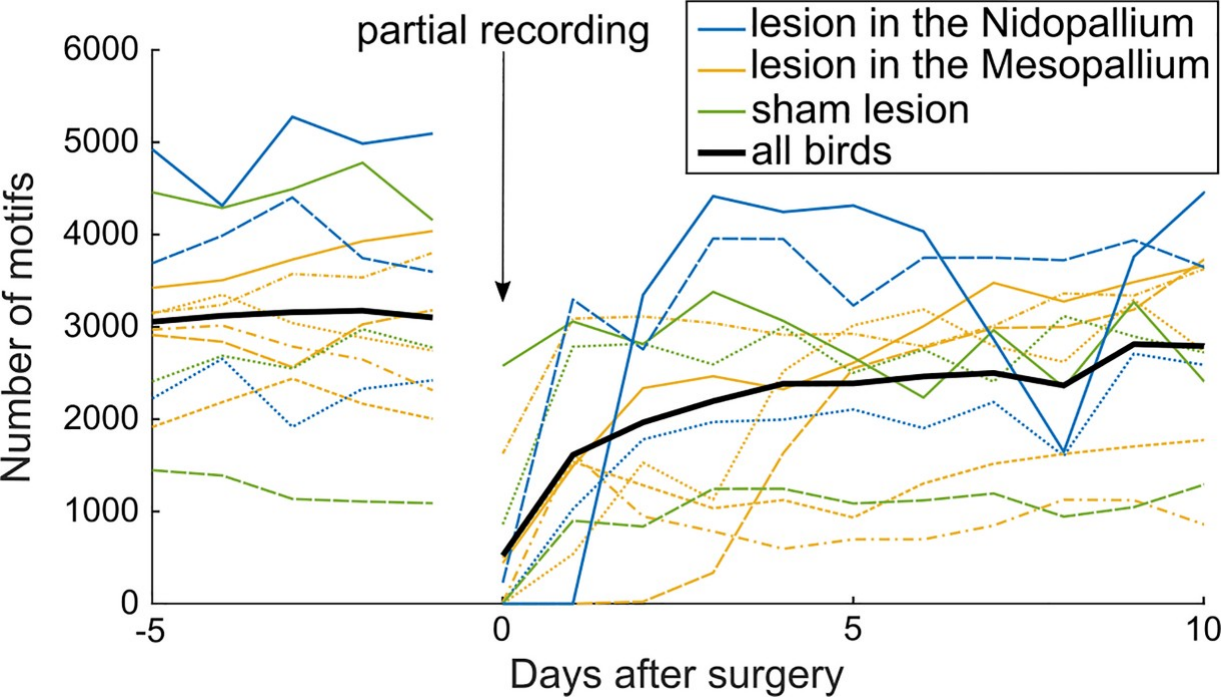
Brain surgeries transiently decrease the singing rate. The number of daily song motifs produced from five days before the surgery up to 10 days after the surgery (N = 12). Surgeries consisted of either an injection of saline (green, N = 3) or ibotenic acid (blue and orange, N = 9) into a brain area outside the known song-control system. On day zero, only recordings from after the surgery were analyzed. Different line styles indicate different birds and the black line indicates the average over all birds.

**Fig 4:**
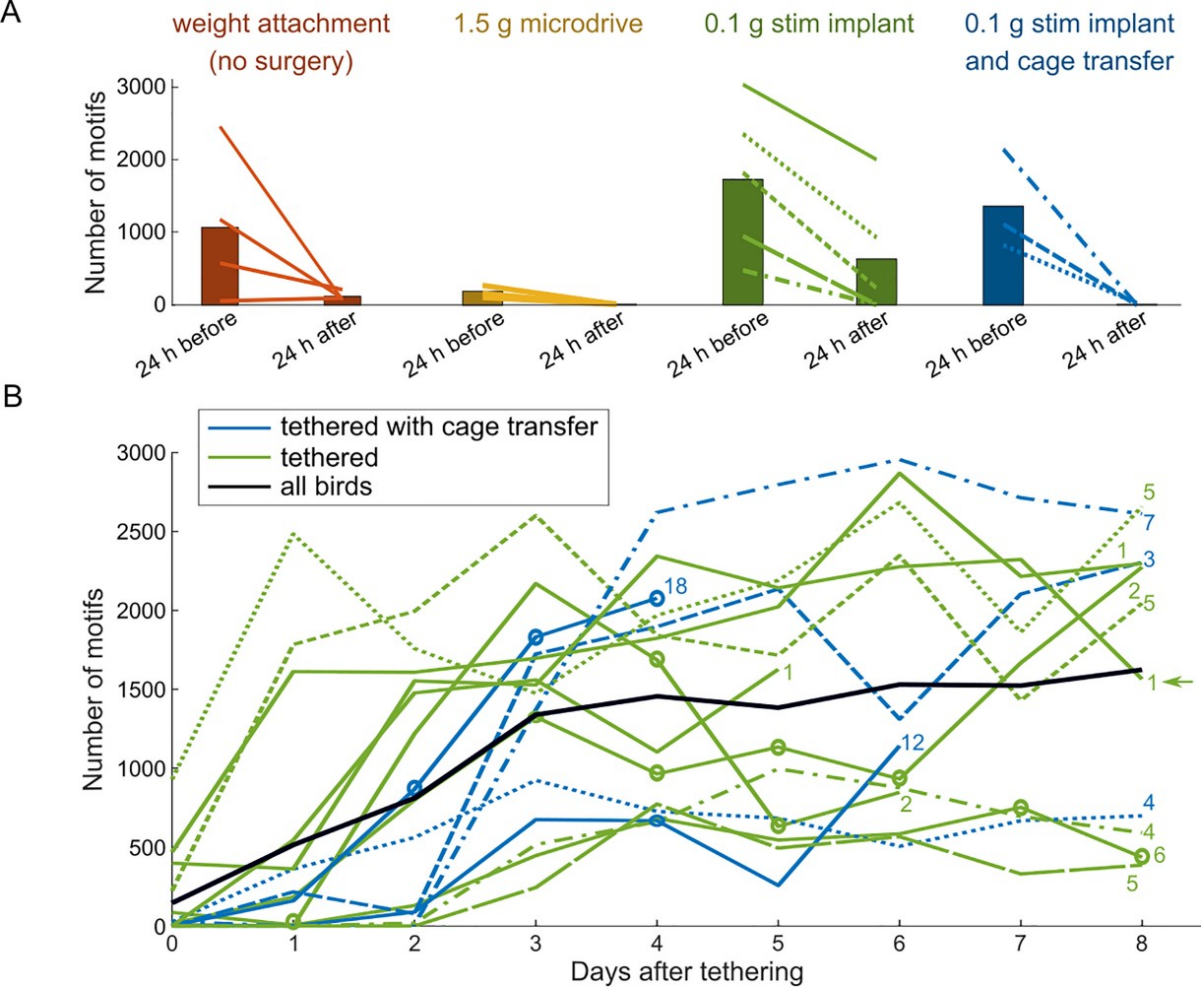
Tethering transiently decreases the singing rate. (A) The number of song motifs produced in the 24 hours before tethering onset (left bar) and the 24 hours after tethering onset (right bar, lines connect individual birds). Birds were either tethered before surgery but after weight attachment (N = 4, weight attachment), after microdrive implantation surgery (N = 5, microdrive) or after stimulation electrode implantation surgery (N = 8, stim implant). Some birds (N = 3, cage transfer) were also transferred into a smaller cage on the same day. All birds were tethered for the first time except the bird that sang around 2000 motif renditions after tethering (in A, stim implant), this bird is marked with a green arrow in panel B where singing rate from the first time tethering 1 day after surgery is shown (see Methods). (B) The number of song motifs increased gradually after the start of tethering on day 0 (day 0 corresponds to the first 24 hours after tethering). The number of days between the surgery and tethering onset is shown for each animal as a (blue or green) number, where green marks tethered-only birds, blue marks birds that also changed the cage on the same day, and black marks the average over all birds. The bird that was tethered 10 days after surgery has been tethered before. Five birds received an LED implant for optogenetics, the circles mark the days with optogenetic manipulations. All other birds received an implant for electrical stimulation. The diverse line styles indicate the corresponding birds in Panel A; solid lines mark birds without data available 24 hours before tethering.

Our experiments started by habituating single-housed birds to the cage and the soundproof box for several days. Earlier work indicates that zebra finches adapt to social isolation within a day [16, 39]. We imposed ≥ 5 such adaptation days for: unmanipulated birds (Fig 1), birds with interactive white noise (WN, Fig 2), birds with brain surgery (without implant, Fig 3), and tethered birds (Fig 4) after weight attachment (without surgery); we imposed 2–6 days (before surgery) for birds with 0.1 g stim/LED implant and tethering (Fig 4); and we imposed 1–2 days (before surgery) for birds with a microdrive implant and tethering (Fig 4). We report the dynamics of the singing rate during the adaptation process in unmanipulated birds in Fig 1A.

All birds were monitored daily to evaluate their overall health using the following parameters: body posture, motor activity, alertness, and eating and drinking behavior. In addition, all birds that underwent surgery were examined 1 h, 2 h, and the next morning after the surgery. Sentinel birds in our colonies were free of parasites and were tested negative for bacterial pathogens.

### Song recording and analysis

Songs were recorded with a wall-attached microphone (Audio-Technica PRO 42, Audio-Technica, Tokyo, Japan), digitized to 16 bits at a sampling rate of 32 kHz, and analyzed with custom scripts implemented in MATLAB (MathWorks, Inc, Natick MA, United States). To visualize the songs, we computed log-power sound spectrograms, which are time-frequency representations of sound intensity.

To count the number of motifs, we selected an easy-to-classify syllable that was present and singly produced (not repeated) in each song motif. Semi-automatic detection of a target song syllable was performed by a two-layer neural network. To train the neural network, one for each animal, we first visually inspected spectrograms and labeled a small number of song syllables, calls, and noises (e.g. wing flapping) from the first few days of the experiment. The neural network was then trained to detect the target syllable (or a part of this syllable). To reduce false-positive detections, our syllable detector rejected syllables with pitch (or duration, depending on the experiment performed) outside the range (> 5 standard deviations) of the targeted syllable. We visually inspected the automatically detected syllables and discarded false positive detections. In randomly selected 10 birds from Fig 2F, on the first 16 days following habituation, we found at most 1% false-positive detections per day. We did not attempt to manually correct false negatives because their identification was too laborious (in previous work, we found false negatives to account for 0.6% of automatic detections on average [35]).

### Baseline group of isolated birds

To count song motif numbers in isolated birds (Fig 1), we selected data from 19 birds used for Chapters 2–4 of a Ph.D. Thesis [40]. All birds were never subjected to surgery nor a behavioral manipulation and were singly housed for at least 14 days (at least 9 days after a 5-day habituation period).

To inspect the stability of the singing rate in this bird group after a long period of isolation, among these 19 birds, we randomly selected 6 birds and re-analyzed their singing rates at the beginning of the isolation periods. To visualize the gradual increase in singing rate at the beginning of isolation, an exponential curve was fitted to the daily number of motifs excluding day 0 (partial recording) using the MATLAB function fit (colored lines, Fig 1A). We then tested the singing stability by comparing the (adapted) singing rate on days 11–13 after isolation start to the rate on the last three days of the isolation period (Fig 1B, N = 3 birds isolated for at least 29 days; N = 3 birds isolated for at least 68 days, see legend in Fig 1A). None of the birds were manipulated either on days 11–13 or on the last three days of isolation; however, intermittently, the following manipulations occurred (Fig 1A): the bird labeled in dark blue was in constant dim light for 10 days and was exposed to playback of its own song for 5 days; the birds labeled in red, purple, and green were exposed to white noise (WN) for 8, 10, and 14 days, respectively; the birds labeled in yellow, purple, green, and light blue had a female company outside of their isolation chamber for 5, 8, 9, and 17 sessions, each less than 5 minutes long and distributed across 1, 2, 3, and 3 days, respectively; additionally, the bird labeled in green had once a female company in the same isolation box, but in an adjacent cage for 2 hours and 40 minutes. The last manipulation happened 11 (dark blue), 4 (red), 16 (yellow), 5 (purple, green), and 21 (light blue) days before the end of the isolation period.

### Song complexity

To test whether song complexity changes during social isolation, we counted the syllables in motifs and applied a linear mixed-effect model to the 6 birds in Fig 1A. The two fixed effects were 1) the number of syllables per motif and 2) the change in this number after a long period of isolation. In each bird, we randomly selected 160 song motifs both at the beginning and at the end of the isolation. Individual differences across birds were modeled as random effects.

### Interactive white noise (WN)

We inspected the effects of interactive WN on the singing rate (Fig 2). To this end, we analyzed data from all 26 birds presented in Chapters 2–4 [40]. To selectively reinforce certain renditions of a targeted harmonic syllable, WN (roughly 85 dB at bird’s position) was delivered when the detected syllable feature (pitch or duration) was below or above a manually chosen threshold [35]. On each day, before the lights went on, detection thresholds were adjusted to roughly the median feature value from the previous day (aiming for a 50% hit rate of the targeted syllable).

Two of these 26 birds have also been used to test the singing rate of unmanipulated birds (Fig 1C and 1D) because they were not exposed to WN for the first 14 days after isolation. Note, however, that the three birds from Fig 1A and 1B that were exposed to WN between day 13 and the end of the isolation period were not included in the WN analysis because WN was triggered both on very high and very low-pitched versions of their target syllable. For a more in-depth analysis of the behavioral effect of WN (whether birds were attracted or repelled by WN), we analyzed 25 additional birds as indicated (resulting in 51 birds in total).

To test whether WN affects the singing rate, we compared the minimum number of motifs per bird between WN-exposed birds (on WN days only, N = 26 adults) and unmanipulated birds (N = 19 adults). On average, birds were exposed to WN for 11.3 ± 5.2 days. To match the numbers of days analyzed between these bird groups, we performed a random subsampling procedure: We first randomly selected 19 birds from the 26 WN birds and used their numbers of days in experiment to randomly sub-select equal numbers of days from the 19 unmanipulated birds (if this was not possible, we repeated the sub-selection). This sub-sampling resulted in the inclusion of 11.3 ± 4.6 days on average per unmanipulated bird, closely matching the average 11.3 days of WN exposure.

### Anesthesia and analgesia

All procedures requiring surgery were done under isoflurane anesthesia (Attane, Piramal Healthcare Limited, India) at 3–4% for induction and 0.5–1.5% for maintenance. Before the onset of the surgery, we provided analgesia with the nonsteroidal anti-inflammatory drug carprofen (2–4 mg/kg, Norocarp, ufamed AG, Sursee, Switzerland) that we administered intramuscularly (IM).

After positioning the bird in the stereotaxic apparatus, we applied the skin antiseptic povidone-iodine (Betadine, Mundipharma Medical Company, Basel, Switzerland) to the incision site, followed by the local anesthetic lidocaine in gel form (5%, EMLA, AstraZeneca AG, Zug, Switzerland).

### Brain lesions

We performed bilateral lesions in either the nidopallium or the mesopallium with 500 nl of ibotenic acid (R&D Systems Europe, Ltd., Abingdon, United Kingdom) injected with a micropipette using a Picospritzer III (Parker Hannifin, Hollis NH, United States) [35]. Bilateral sham lesions were performed with 500 nl of Ringer’s solution (B. Braun Melsungen AG, Melsungen, Germany).

### Implantations of microdrives and of stimulation electrodes

To record neural activity, birds were implanted with a custom-made 1.5 g, 16-channel microdrive with carbon fiber electrodes either in the ventral tegmental area (VTA) or in the lateral magnocellular nucleus of the anterior nidopallium (LMAN) part of the song system [41]. After implantation, the awake birds were briefly held in protective foam while we manually adjusted the dorsoventral position of the electrodes to search for neuronal activity.

To perform manipulations of brain activity, we implanted bipolar stimulation electrodes of 50 *μ*m diameter into LMAN [42, 43].

### Optogenetics

Birds were bilaterally injected with 400 nl of AAV9-CAG-ArchT (University of Pennsylvania Vector Core, Philadelphia PA, United States) into HVC (used as a proper name) using a Nanoject II (Drummond Scientific Company, Broomall, PA, United States). Thereafter, a light-emitting diode (LED, 568 nm, LXML-PX02, Lumileds, San Jose CA, United States) was implanted over HVC.

### Tethering

After implantation of an LED (N = 5 birds), microdrive (N = 5 birds), or bipolar stimulation electrodes (N = 10 birds), birds were tethered to a motorized commutator system [44]. The implantation surgery was performed 2–3 days before tethering onset in birds that were implanted with a 1.5 g microdrive, 1–7 days before tethering onset in birds with 0.1 g stimulation electrodes, and 2–12 days before tethering onset in birds with an LED implant. Four birds did not undergo surgery before tethering, instead, these birds were habituated to being tethered (weight attachment) using the following procedure: Across 5 days, we cumulatively added modeling clay in 0.2–0.3 g steps (up to 1.5 g in total) on top of a plastic cap (0.5 g) that was glued to the cranial feathers with cyanoacrylate glue. Birds were then tethered for 1–2 days before implantation surgery.

Some birds (cage transfer, N = 5, blue lines in Fig 4) were transferred to a smaller cage (type 1 to type 2 or 3) upon tethering. All other birds were housed in the same cage (type 2 or 3) before and during tethering.

To compare the singing rates 24-h pre and post tethering (Fig 4A), in total, 17 tethering events were available. In 8 additional tethering events, no data was available 24 hours before tethering. To study the singing-rate dynamics after tethering (Fig 4B), there were 15 tethering events available that included data from the subsequent 3+ days on which no additional manipulation occurred; in all other cases, the singing rate analysis was not possible because birds either underwent surgery (after weight adaptation and tethering) or they were subjected to handling (following microdrive implant).

Two birds shown in Fig 4 have been tethered twice. For one bird, data was available from after the second tethering event (bird tethered 10 days after surgery, Fig 4B). For the other bird, there was data from after the first tethering event (tethered 1 day after surgery, marked with an arrow in Fig 4B) and from 24-h pre/post the second tethering event (tethered 72 days after surgery, Fig 4A), but not from any other days.

### Bootstrapping welfare thresholds

The meaning of the welfare threshold is to consider birds that sing fewer song motifs per day than this threshold as stressed. We bootstrapped welfare thresholds from our song data in 19 unmanipulated birds (Fig 1C). From these birds, we randomly drew 100’000 birds (with replacement) and from each of them, we randomly drew 10 days after the habituation period (with replacement) to calculate the 5%, 1%, and 0.5% percentiles of singing rate that we termed the welfare thresholds W_95_, W_99_, and W_99.5_, respectively. For example, if we use the threshold W_99_, we would mistakenly conclude that on 1% of the days in Fig 1C, birds were exposed to a stressor.

### Bayesian analysis

We used Bayes’ theorem to estimate the posterior probability *P*(*m* = 1|*s*) of a recent stressful manipulation (*m* = 1) given the current singing rate *s*:

$$P(m=1|s)=\frac{P(s|m=1)P(m=1)}{P(s|m=1)P(m=1)+P(s|m=0)P(m=0)}.$$

**Fig 5:**
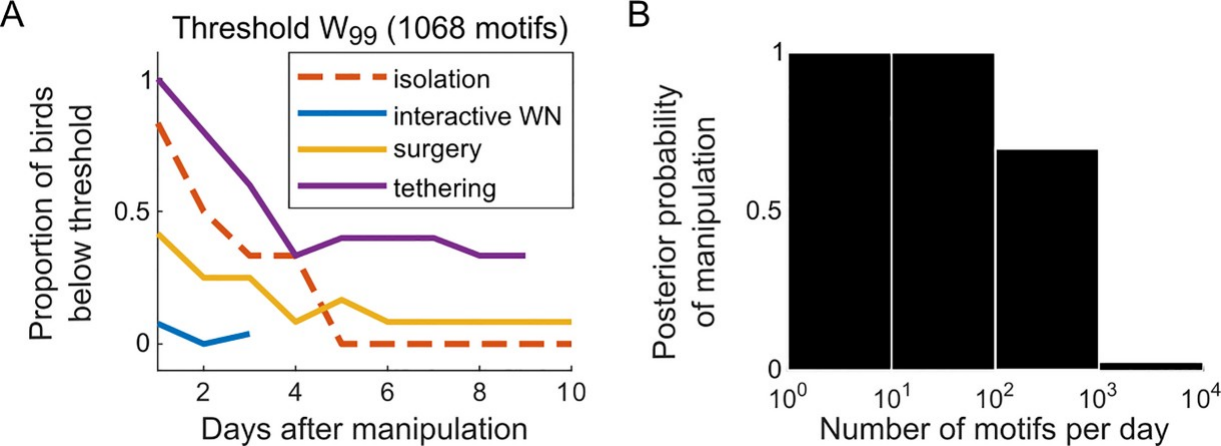
A welfare threshold on singing rate and the posterior probability of manipulation. (A) The proportion of birds not having fully recovered from the manipulation as a function of time since onset of the manipulation for interactive WN (WN, blue, N = 26, Fig 2B), surgery (yellow, N = 12, Fig 3), and tethering (purple, N = 15, Fig 4E), i.e., the proportion of birds with a singing rate below the welfare threshold W_99_. For illustration, also shown are birds subjected to isolation (dashed red, N = 6, Fig 1A). (B) Posterior probability of a recent manipulation (here tethering), as a function of the singing rate.

We estimated the likelihood *P*(*s|m* = 1) of the singing rate following a manipulation by combining the data from one day after surgery (N = 12 birds, Fig 3) and from 24 hours after first tethering (N = 23 birds, Fig 4, two tethering events were excluded because that bird has been tethered before). We then estimated the likelihood *P*(*s|m* = 0) of the singing rate *s* given no recent manipulation (*m* = 0) from data in unmanipulated birds (Fig 1C). In total, there were *N*_*m* = 1_ = 35 data points (days) with manipulation and *N*_*m* = 0_ = 315 data points (from 19 birds) without manipulation. We expressed the prior probability *P*(*m*) of a manipulation as $P(m)=\frac{N_{m}}{N_{m=1}+N_{m=0}},$ for *m* = {0,1}. The posterior probability of a manipulation *P*(*m* = 1|*s*) is shown in Fig 5C, after we discretized the singing rates *s* into four logarithmically distributed bins with borders 10^*i*^ (*i* = 0,…4).

Because we found no significant difference in singing rate between birds without a manipulation and birds with WN exposure, we also estimated *P*(*s|m* = 0) by combining the data from all these 43 birds (Fig 2F), resulting in *N*_*m* = 0_ = 1087 days without manipulation. Given this additional evidence, the posterior probability of a manipulation was more binary-like, it was either equal to one below a threshold value or to zero above that value. Thus, the well-known trend that ‘more data yields higher certainty’ applies also to the estimated presence of a recent stressor.

### Euthanasia

At the end of the experiments, most birds were returned to the bird colony. However, birds that had undergone surgery were euthanized with an overdose of intraperitoneal injection of sodium pentobarbital (200 mg/kg, Esconarkon, Streuli Pharma AG, Uznach, Switzerland).

### Statistics

In all experiments, the experimental unit is the bird (N). All statistical tests were performed at a significance level of ɑ = 0.05. We report absolute numbers of motifs (per day) and their standard deviations. When we compared several data points in each bird before and after manipulation, we tested whether the mean motif count after the manipulation significantly differed from the mean motif count before the manipulation using a paired two-tailed t-test. We obtained qualitatively identical results when we tested whether the data points after the manipulation (in percent) significantly differed from 100%.

## Results

We analyzed the singing rate (number of daily song motifs, unless specified) by focusing particularly on its stability and suitability for serving as a baseline against which to compare the effects of experimental manipulations. All data analyzed stemmed from earlier experiments—some of which are published [35, 36]—aimed either at probing the plasticity of song syllables in terms of their pitch/duration or at studying/manipulating neuronal activity. The goals of these experiments and the planned/performed analyses were not linked with either welfare or singing rate as a behavioral readout. For this reason, a human bias in reported song counts is unlikely.

### Stable baseline singing rate and high song complexity in isolated male zebra finches

In many experiments, zebra finches are individually housed for various reasons such as to ensure high-quality song recordings, to control the sensory and social environment, and to enable inferences about causality. When we isolated adult male zebra finches (N = 6) from our colony in a new environment (cage type 1), the average singing rate was 746 ± 1074 motif renditions on the first day (range: 0 to 2792 renditions). Afterward, the singing rate increased to 2906 ± 1388 renditions on average on day 13 (range: 1428 to 5170 renditions, Fig 1A). Thereafter, the singing rate remained relatively stable for up to 88 days (P = 0.16, t5 = -1.63, paired two-tailed t-test between an average of 2621 ± 1191 motifs per day across the last three days of the experiment and an average of 3045 ± 1495 motifs per day across days 11–13, Fig 1B).

In experiments in a larger cohort, we found that isolated zebra finches, after a habituation period of at least 5 days, reliably sang at high rates of 2818 ± 1183 motifs per day (315 days analyzed from N = 19 birds, Fig 1C). In these birds, we also assessed the spontaneous fluctuations in singing rate (i.e., the ‘noise’ opposing the signal) and found that none of the birds produced fewer than 629 undirected song motifs per day, which was the minimum we found across all birds and days (Fig 1C inset and 1D). We did not find a correlation between a bird’s age at the beginning of the analyzed period and the minimum number of song motifs produced (R = -0.08, P = 0.75, N = 19 birds, age = 205 ± 169 dph, minimum 101 dph, maximum 815 dph). Results did not significantly change when we excluded the oldest bird (R = -0.05, P = 0.86, N = 18 birds, age = 171 ± 84 dph, minimum 101 dph, maximum 376 dph) or when we took the mean instead of the minimum number of song motifs (R = -0.02, P = 0.94, N = 19 birds, age = 205 ± 169 dph, minimum 101 dph, maximum 815 dph).

Isolated adult zebra finches change the acoustic features of distance calls [38]. Therefore, besides singing rate, isolation could also affect song complexity, i.e. change in the number of song syllables per motif. To analyze the song complexity data (see Methods), we applied a linear mixed-effect model and found that a prolonged period of isolation resulted in a small but significant increase in song complexity of 0.13 ± 0.04 syllables per motif (P = 6*10^−4^, linear mixed-effect model, t1598 = 3.42).

Our findings show that the singing rate in isolated male zebra finches is a very robust parameter that reliably exceeds some minimum value each day. In the following sections, we assess whether deviations caused by experimental manipulations could be easily detectable.

### Interactive white noise (WN) does not affect the singing rate

Altering auditory feedback during song production induces changes in song structure, which can be exploited to study reinforcement learning and sensorimotor integration. For example, zebra finch song can be operantly conditioned using loud acoustic stimuli played contingent on sound features such as pitch (fundamental frequency) or syllable duration. In such experiments, birds tend to adapt their songs to avoid the syllable variants that trigger the stimuli [45, 46].

Here, we tested whether the use of WN as an acoustic stimulus affects the singing rate. We delivered noise stimuli of 50 ms duration contingent on the pitch or duration of a song syllable (Fig 2A). We expected birds to avoid such stimuli by singing less. However, we found no decrease in the number of song motifs on the first day relative to the number of motifs on the day before (ratio 1.01 ± 0.29, paired two-tailed t-test: t25 = -0.13, P = 0.90, N = 26 birds, Fig 2B). We further tested whether WN had a more transient effect, which could have been averaged out in our day-by-day analysis. But even within the first hour after exposing birds to WN for the first time, the number of target syllables was stable relative to the matched hour on the day before (relative change 0.97 ± 0.55 (SD), paired two-tailed t-test: t24 = 1.04, P = 0.31, one bird excluded because there were no songs during the time-matched hour on the day before WN). Furthermore, in addition to the stability of singing rate, none of the birds visibly changed the spatiotemporal structure of their song motifs.

When comparing the average singing rates on days with and without WN, we found no statistical difference (the ratio of average motif counts was 1.05 ± 0.16, t25 = -1.59, P = 0.12, N = 26, two-tailed paired t-test, Fig 2D, Fig 2E). Furthermore, we did not find a significant difference between the minimum number of motifs per bird in WN birds (days with WN only) and in unmanipulated birds in time-matched periods since start of isolation (P = 0.86, stat = -0.17, N = 26 birds with 11.3 ± 5.2 WN days per bird and N = 19 unmanipulated birds with 11.3 ± 4.6 days per bird, Wilcoxon rank-sum test, see Methods).

In most (40/51) birds, WN nevertheless had a behavioral effect in that these birds shifted the targeted syllable feature away from the noise-triggering region (P < 0.01 according to the z-score criterion in [35]). Interestingly, among the remaining 11 birds, 9 maintained the targeted sound feature and 2 responded by singing more noise-triggering syllable renditions (Fig 2C). In these latter two birds, the fraction of syllables triggering WN was higher late in the day compared to early in the day (first 200 song motifs on a day: 55 ± 3% vs. the last 200 song motifs on the same day: 76 ± 3%, one-tailed paired t-test, t24 = 6.49, *P* = 5*10^−7^; 25 days). One of these two birds was exposed to WN when a song syllable was shorter than a daily fixed threshold, the other was exposed to WN when the pitch of a syllable was below a threshold.

### Brain surgeries transiently suppress the singing rate

Brain lesions are a useful means to investigate the functional role of a brain region. When we determined whether brain lesions affected the singing rate, we only inspected lesions in the nidopallium or the mesopallium, which are brain areas outside of the classical song control system, to make sure the singing rate was not directly affected by a neurological impairment, but rather by the burden of the manipulation, i.e. the surgery. After the lesions (on day 1), the average singing rate decreased to 54 ± 34% compared to the average on the 5 days before the lesions (P = 0.004, paired two-tailed t-test: t11 = 3.68, range: 0–104%, Fig 3). Ten days after the surgery, the singing rate recovered to 91 ± 26% of the average rate before the surgery (paired two-tailed t-test: t11 = 1.37, P = 0.20, range: 31–128%).

### Tethering transiently reduces the singing rate

To record neuronal activity or to stimulate the brain, isolated male zebra finches are usually tethered to a motorized commutator system [44] which counteracts animal rotation and allows the birds to move freely in the cage, albeit with an increased mass and torque on the head. We found that tethering initially reduced the singing rate in all four groups of birds studied, Table 1, Fig 4A. In the interval 24 h after the start of tethereing, the number of song motifs produced decreased to 22%±43% compared to the average number of motifs produced in the same interval just before tethering (combining data from all four groups, two-tailed t-test, t16 = -7.55, P = 1.2*10^−6^).

**Table 1:** Singing rate 24 hours before and 24 hours after four distinct manipulations involving tethering.

Manipulation	# motifs, 24-h before (mean±std, range)	# motifs, 24-h after (mean±std, range)	Percent decrease
Weight attachment, N = 4	1066±1033, 56–2455	117±65, 67–212	45 ± 79%
Microdrive implant, N = 4	189±79, 94–281	9 ± 13, 0–27	95 ± 8%
Electrode implant, no cage transfer, N = 5	1736±1041,478–3050	634±859, 0–2010	45 ± 79%
Stim. electrode implant, cage transfer, N = 3	1369±699, 829–2158	9±5, 0–26	99 ± 2%
Overall, N = 16	1069±965, 56–3050	218±514, 0–2010	78 ± 43%, 0–171%

After tethering onset, the singing rate increased gradually (Fig 4B). After five days of tethering, the singing rate was at 1430 ± 795 motifs per day (in one bird, in which no data from the fifth day of tethering was available, we used data from the fourth day instead). The singing rate after five days of tethering was significantly lower than the rate five days after surgery only (2387 ± 1045 per day, two-tailed t-test, t25 = -2.70, P = 0.01) and the rate after five days of isolation (2532 ± 1480 per day, two-tailed t-test, t19 = -2.23, P = 0.04). Note that the singing rates pre- and post-tethering were likely affected by a cumulative burden that, in addition to tethering, included the implant’s weight on the head. However, we did not find a significant difference between birds with a relatively heavy implant (≥ 1 g, microdrive, and weight attachment, relative singing rate change one day pre/post tethering onset: 0.85 ± 0.25) and birds with a relatively light implant (0.1 g, relative singing rate change 0.73 ± 0.55, two-tailed t-test T15 = 0.57, P = 0.58).

### Singing rate as a welfare threshold

Our findings suggest that the singing rate can reflect the welfare of zebra finches. The justification of our proposal is rooted in contrapositive logic: if A) putative stressful manipulations lead to a strong reduction in singing rate, and B) in the absence of a stressor (beyond isolation) there are no comparable reductions of singing rate, then, frequent singing implies the absence of stress or the successful coping with stress. To turn this idea into a quantifiable assessment of welfare that is directly applicable in experiments, we defined the concept of welfare threshold.

The threshold W_x_ corresponds to the number of motifs per day that is exceeded by putatively unstressed isolated zebra finches in x percent of cases. Here, x is the confidence level of the threshold, e.g. for x = 99%, we interpret a singing rate below the W_99_ threshold as a 1% probability that a bird is diagnosed as stressed even though there is no acute stressor present.

Using this definition, we used bootstrapping to compute a set of welfare thresholds for x = 95%, 99%, and 99.5% (see Methods, Bootstrapping welfare thresholds). Using data from the 19 isolated birds in Fig 1C/ Fig 1D, we obtained W_95_ = 1335 motifs, W_99_ = 1068 motifs, and W_99.5_ = 765 motifs.

In Fig 5, based on the W_99_ threshold of 1068 motifs, we plot the fraction of birds in which singing rate signals the presence of a recent manipulation (isolation, interactive WN, surgery, and tethering) as a function of the days since the manipulation onset. Fig 5A suggests that after one week, most birds have coped with the manipulations, except for 1/26 birds with interactive WN, 1/12 birds after surgery, and 6/15 tethered birds (Fig 5A).

### Bayesian inference of a recent manipulation

As an alternative to the welfare threshold, we performed a Bayesian analysis to estimate from the singing rate the posterior probability that a stressful manipulation (such as tethering) has recently occurred. We used for this analysis the baseline singing data and the tethering data (the first day since tethering onset, Fig 4, see Methods). We found that the posterior probability of a recent manipulation equaled one below 100 motifs per day, it was about 0.7 between 100 and 1000 motifs per day, and above 1000 motifs per day it was essentially zero (Fig 5B). Hence, Bayesian inference informs us that the singing rate is an excellent behavioral variable to infer the presence of a recent putative stressor such as tethering.

### Fully automated quantification of the singing rate

To evaluate whether welfare monitoring can be performed using fully automated procedures (not involving human inspection), we counted song motifs by performing for each manipulation (isolation, surgery, and tethering interactive WN—one animal each) automatic detection of syllables (without manually discarding the false positive detections, see Methods).

We found that all inspected birds crossed the three welfare thresholds (W_95_, W_99,_ and W_99.5_) on the same day as with manually corrected song numbers. These findings lend support to the idea that an automatic welfare assessment using automatic song counting is feasible.

## Discussion

We found that diverse experimental manipulations such as brain lesion and tethering threaten a bird’s homeostasis [19], as evidenced by the associated transient decrease in the singing rate. The fact that birds reliably reduced the singing rate following these manipulations makes it possible to infer the existence of a preceding manipulation from observation of a low singing rate. This state of affairs is informative about welfare insofar as the applied manipulations were stressful to the birds. If either tethering or brain lesions should be considered non-stressful manipulations, then their effects on the singing rate must be due to something else than stress. However, except for interactive WN, the tested manipulations have insulting effects on free behavior or normal body function (e.g. hypothermia and loss of protective reflexes during surgery). Therefore, a non-stressful nature of these manipulations seems unlikely and we conclude that reduced singing rate is indicative of the presence of a stressor.

Interactive WN is the only manipulation examined that was not associated with less singing (Fig 2). In this regard, we have to emphasize that we only tested WN of roughly 85 dB sound intensity, so we cannot discard that a louder noise stimulus could affect the singing rate. Our observation that interactive WN at the chosen intensity is not a reliable stressor agrees with findings that such stimuli can be appetitive reinforcers of place [47].

Although we did not inspect any welfare parameter other than singing rate, this single parameter perfectly discriminates tethered birds with a relatively heavy implant from unmanipulated birds. Thus, it follows that no additional welfare readout was necessary for inferring the presence of the tethering manipulation. Hence, to monitor additional welfare parameters such as body weight or corticosterone would not alter the fact that reduced singing rate by itself is highly indicative of the presence of this type of putative stressor (corticosterone would likely signal the same information given its correlation with vocalization rate [48]).

That singing rate is a reliable read out of a recent putative stressor is also supported by the effect of other stressors such as restricting the availability of food and water [33] and mounting a backpack [34], both of which negatively impact the singing rate. In combination, there is ample evidence that singing rate can serve as a non-invasive measurement of welfare in isolated male zebra finches.

We proposed a set of welfare thresholds for laboratory populations of zebra finches. These thresholds are rooted in the robustness of the left tail of the singing rate distribution in unmanipulated birds (no unmanipulated bird produced less than 600 song motifs after the habituation period, Fig 1C). The welfare threshold reflects the behavior of putatively unstressed birds. How to choose the percentile of the welfare threshold in practice depends on the level of optimism about stress assessment. For example, if one believes that birds were indeed stress-free on 100% of baseline days, one will choose the hundredth percentile and the threshold, i.e., W_100_. A more pessimistic researcher, believing that 5% of birds in the baseline population were stressed for whatever unknown reason despite their avid singing, will choose the level W_95_. The lower the chosen percentile, the higher the threshold on singing rate, and the more promptly a stressor will be inferred based on the chosen welfare threshold.

Working with the welfare threshold W_99_, we conclude that surgeries caused stress on the next day in 5/12 animals (Fig 3), and tethering caused stress in 24/24 animals (Fig 4). Birds tended to recover their high pre-manipulation singing rate within a few days, based on which we infer that their coping capacity is generally high for the tested manipulations. The only manipulations that chronically suppressed singing for more than 3 days were: one brain-lesioned bird that remained below W_99_ for 6 days and 5 tethered birds that remained below W_99_ for at least 8 days. One tethered bird increased its singing rate quickly above W_99_, but on the fifth day, it decreased it below W_99_, suggesting that the drop in the singing rate was no longer an acute response to tethering.

As a future tool, one possible application of the welfare threshold could be to minimize the possibility of a cumulative burden, which is to wait until the singing rate exceeds W_99_ before starting any subsequent manipulation. Indeed, note that some birds were below the W_99_ threshold before tethering onset; thus, in these birds, the stress from surgery or from cage transfer and handling should be considered as cumulative. This being said, we tethered only very few birds with relatively light implants (0.1 g) without cage transfer (green birds in Fig 4), which is why the effect of tethering such birds should be assessed again in the future using a larger cohort of birds. Other useful applications of our methods could be to timely adopt stress-easing measures such as to remove the tether, to provide a temporary companion, to provide weight relief, or to abort the experiment.

As an alternative method to translate our observations into practical numbers, we have also computed the posterior probability of a putative stressor using Bayesian inference. In practice, the posterior probability could, for example, be thresholded to reach a decision about stress-easing measures to adopt. Simple thresholding thus seems to be a computationally simple and adequate approach to assess welfare based on singing rate. Likely, both methods will provide similar answers and the one to be used is a matter of personal taste.

The main challenge of applying our measures is to count the hundreds to thousands of songs per day, which however can be remedied by our unbiased and automated proof-of-concept approach. Because singing rate measurements are contact-free, rapid, and is relatively easy to implement, their inspection may lead to refinement on the available welfare monitoring methods in birds [24, 49, 50]. Establishing welfare thresholds for the most commonly studied songbird would have a great impact when studying the mechanisms underlying the control and plasticity of communication.

One drawback of using the singing rate as an indicator of welfare is that it does not apply to non-singing female zebra finches. However, in males, not only the singing rate but also the calling rate recovers after acute manipulations (e.g. backpack attachment) [34, 51], suggesting that the calling rate could also serve as a more general indicator of welfare [52]. Thus, we see no major obstacle in trying to generalize our approach to female zebra finches and other animals, including non-vocal learners [53, 54].

Although our study was not designed to be informative about the welfare of isolated animals, it can provide some clues. In parrots, isolation causes chronic stress that can lead to self-mutilation, feather picking, and excessive screaming [55]. However, in over 15 years of experimenting with isolated zebra finches (on the order of 1000 birds), we have never observed any of these maladaptive behaviors. Moreover, completely isolated juveniles learn excellent song copies from song playbacks through a loudspeaker [56], which demonstrates the high adaptability in the isolated state. In future work, it will, therefore, be important to evaluate the effect of social isolation on the singing rate in experiments in which pre-isolation singing data is available. Similarly, it will be interesting to evaluate the interplay between stressors and singing rate also in social conditions, to verify whether a similar welfare-signaling role can be conferred to it. For example, one might want to test whether stress resulting from visually confirmed conflicts or fights between animals is associated with a transient reduction in singing rate.
